# Prickly Pear Betalain-Rich Extracts as New Promising Strategy for Intestinal Inflammation: Plant Complex vs. Main Isolated Bioactive Compounds

**DOI:** 10.3389/fphar.2021.722398

**Published:** 2021-09-14

**Authors:** A. Smeriglio, C. De Francesco, M. Denaro, D. Trombetta

**Affiliations:** ^1^ Department of Chemical, Biological, Pharmaceutical and Environmental Sciences, University of Messina, Messina, Italy; ^2^ Foundation Prof. Antonio Imbesi, University of Messina, Messina, Italy

**Keywords:** *Opuntia ficus indica* L. (Mill), prickly pear, betalains, antioxidant activity, anti-inflammatory activity, Caco-2 cells

## Abstract

Recently, many studies have highlighted the health effects of betalains beyond their use as food dyes. The present study investigated betalain-rich extracts with different colors and their main bioactive compounds in order to provide first evidence as a new promising strategy for intestinal inflammation management. Prickly pear betalain–rich extracts, obtained by a QuEChERS method, have been characterized by LC-DAD-ESI-MS/MS analysis. The potential role of betanin, indicaxanthin, and prickly pear extracts in counteracting the antioxidant and anti-inflammatory events was evaluated by several *in vitro* cell-free and cell-based assays. Indicaxanthin and betanin represent the most abundant compounds (≥22.27 ± 4.50 and 1.16 ± 0.17 g/100 g dry extract, respectively). Prickly pear extracts showed the strongest antioxidant and anti-inflammatory activities with respect to the pure betalains both on *in vitro* cell-free and cell-based assays, demonstrating the occurrence of synergistic activity, without any cytotoxicity or alteration of the barrier systems. The release of reactive oxygen species (ROS) and key inflammatory markers (IL-6, IL-8, and NO) was strongly inhibited by both betalains and even more by prickly pear extracts, which showed a similar and sometimes better profile than the reference compounds trolox and dexamethasone in counteracting the IL-1β–induced intestinal inflammation.

## Introduction

Betalains are water-soluble, tyrosine-derived pigments characterized by a common chromophore, betalamic acid (Timoneda et al., 2019). These secondary metabolites comprise two classes of compounds, betaxanthins (yellow-orange) and betacyanins (red-purple) (Aruwa et al., 2018). The first ones are derived from betalamic acid via conjugation with different amines and amino acids, whereas the second ones are derived by condensation of betalamic acid with cyclodihydroxyphenylalanine (cyclo-DOPA) (Timoneda et al., 2019). Indicaxanthin and betanin (betanidine-5-*O*-β-glucoside), a betaxanthin and betacyanin, respectively, are the most representative compounds belonging to the betalain family (Belhadj Slimen et al., 2017). These compounds, which confer a typical color to flowers, fruits, and roots, play a pivotal role in the attraction of pollinators, in photoprotection, in counteracting the herbivores’ attack, and in conferring tolerance to drought and salinity stress (Jain et al., 2015). Moreover, by acting as osmolytes, they can increase the plant resistance to pathogenic microorganisms (Esatbeyoglu et al., 2015; Khan and Giridhar, 2015).

Most families belonging to the Caryophyllales order are betalain-pigmented. Although they are characterized by the presence of other flavonoid-derived compounds, such as proanthocyanins, anthocyanins have never been detected in these plant species, drawing a clear pattern of mutual exclusion (Timoneda et al., 2019).

Thanks to their particular coloring and stability at pH values between 4 and 7, betalains are attracting increasing attention as dyes and additives in low-acid foods (Gandìa-Herrero and Garcìa-Carmona, 2013; Cejudo-Bastante et al., 2016). However, beyond their technological use, recently, many studies have highlighted the health effects of these molecules as promising antioxidant, anti-inflammatory, hepatoprotective, antitumor, antidiabetic, hypolipidemic, antimicrobial, radioprotective, and antiproliferative agents (Kaur et al., 2018). However, these investigations are mainly focused on pure synthetic or isolated molecules, and studies on plant complexes rich in betalains are rather lacking.

Recently, our research group has demonstrated that betalain extracts from pulp and peel of prickly pears possess strong antioxidant, cytoprotective, and antiangiogenic activities (Smeriglio et al., 2019), opening the way also to a potential reuse, in a circular economy perspective, of fruit processing waste, in particular the peel, as a rich source of these bioactive compounds. Moreover, in addition to the higher concentration of these bioactive compounds in comparison with the most used source, beetroot (Paz et al., 2015; Smeriglio et al., 2019), prickly pear has several advantages, including the absence of geosmin and pyrazine responsible for various technological and sensory concerns (Rodriguez-Amaya, 2019). Considering these promising results and postulating that these extracts could be potential candidates for developing anti-inflammatory remedies with particular emphasis on intestinal inflammation, we decide, in the present study, to investigate the antioxidant and anti-inflammatory activities of prickly pear pulp and peel extracts, also comparing the biological activity of these plant complexes with their main bioactive compounds (betanin and indicaxanthin).

## Materials and Methods

### Chemicals

Analytical grade chemicals, LC-MS grade solvents and acids (formic acid and acetic acid), and the betanin reference standard were purchased from Merck KGaA (Darmstadt, Germany).

### Sample Preparation

The prickly pears (*Opuntia ficus-indica* (L.) Mill. cv. sanguigna and surfarina) were harvested in Messina (Sicily, Italy) in August 2019. The fresh fruits, ten for each color, red, orange, and yellow, were peeled, and the seeds were manually removed. Fruit peel and flesh were powdered with liquid nitrogen by a blade mill (IKA^®^ A11 basic analytical mill) in order to block the enzymatic activity and to preserve the organoleptic and nutritional properties. The prickly pear peel and flesh betalain extracts (PE and FE, respectively) were obtained by a QuEChERS method according to Smeriglio et al. (2019).

In brief, 20 ml of methanol/water mixture (90:10, *v*/*v*) was added to 10 g of each sample and vigorously shaken for 3 min. Then, 1 g Na_3_C_6_H_5_O_7_, 0.01 g C_6_H_6_Na_2_O_7_ · 1.5 H_2_O, 1 g NaCl, and 4 g MgSO_4_ were added. The mixture was vigorously shaken for 3 min and centrifuged for 20 min at 3,500 × g. After that, the supernatant was transferred to a new centrifuge tube filled with 0.3 g strong anion exchanger (SAX) and 1.8 g MgSO_4_. The tube was shaken for 3 min and then it was centrifuged for 5 min at 3,500 × g. The supernatant was evaporated, and the obtained dry extract was stored in burnished vials with nitrogen headspace until the subsequent analyses. At the time of the analyses, stock solutions of PE and FE in methanol were prepared and properly diluted in 15% CH_3_OH + 0.05% HCOOH.

### Determination of Betalainic Profile by LC-DAD-ESI-MS/MS Analysis

The quali–quantitative determination of betalains was carried out by high-performance liquid chromatography coupled with a photodiode array detector and tandem ion trap mass spectrometry (Agilent Technologies, Santa Clara, CA, United States) (LC-DAD-ESI-MS/MS). An ESI source operating in the positive ionization mode according to the method of Smeriglio et al. (2019) was used. The chromatographic separation was obtained using a Luna Omega PS C18 column (150 × 2.1 mm, 5 μm; Phenomenex), thermostated at 25°C, and as mobile phase, 0.1% formic acid (solvent A) and acetonitrile (solvent B) were used according to the following elution gradient: 0 min, 0% B; 20 min, 20% B; 30 min, 100% B; 35 min, 100% B; 50 min, 0% B; and 60 min, 0% B. The flow rate and injection volume were 0.4 ml/min and 5 μl, respectively.

UV-Vis spectra of betalains were recorded from 190 to 600 nm. Nitrogen was used as dry gas (12 L/min, 70 psi), and nebulizer temperature was set at 365°C. Helium was used as collision gas (4.1 × 10^−9^ bar). Mass spectra were acquired using a fragmentation energy of 1.2 V (MS/MS). Chromatograms were acquired at 477 and 538 nm for betaxanthins and betacyanins, respectively.

Peaks were identified by comparing the retention time, UV-Vis, and mass spectra with those reported in the literature, as well as with pure commercially available standard betanin and isolated indicaxanthin (see *Indicaxanthin Isolation*). Quantification was carried out by external standard calibration curves (1.562–100 μg/ml), expressing the results as grams (g) of each betalain/100 g of the dry extract (DE).

The quantification of the other minor betalains was carried out by extrapolating the data from the calibration curves of the structurally more similar compounds (betanin for betacyanins and indicaxanthin for betaxanthins). Results were expressed as grams of betanin or indicaxanthin equivalents (BE or IE, respectively)/100 g of DE.

### Indicaxanthin Isolation

The isolation of indicaxanthin was carried out by molecular exclusion chromatography starting from the indicaxanthin-rich sample (orange FE), according to the method developed by Tesoriere et al. (2014), with some modifications. In brief, a 30 × 3-cm chromatographic column was packed with 35 g of Sephadex G-25. The orange FE (4 g) was dissolved in a mixture consisting of 3 ml CH_3_OH/15% CH_3_OH with 0.05% CH_3_COOH (50:50, *v*/*v*) and eluted with a flow rate of 3 ml/min, using 1% CH_3_COOH as the mobile phase. The obtained fractions were first analyzed by a UV-Vis spectrophotometer at 477 and 538 nm (maximum absorption wavelengths of betaxanthins and betacyanins, respectively) and subsequently by LC-DAD-ESI-MS/MS analysis to identify those with the highest content of indicaxanthin. The selected fractions were collected in a round-bottom flask and dried by using a rotary evaporator (Buchi R-205, Cornaredo, Milano, Italy). The dry fraction was stored in a burnished vial with nitrogen headspace and then analyzed by LC-DAD-ESI-MS/MS to evaluate the indicaxanthin purity degree.

### *In vitro* Cell-Free Assays

The antioxidant and anti-inflammatory activities of red, orange, and yellow PEs and FEs were evaluated by colorimetric *in vitro* cell-free assays based on different reaction mechanisms and environments. Absorbance data, recorded by a UV-Vis spectrophotometer (Shimadzu UV-1601, Kyoto, Japan) against a blank consisting of methanol, instead of the sample, were expressed as half-maximal inhibitory concentration (IC_50_, μg/ml) with confident limits (CLs.) at 95% calculated by the Litchfield and Wilcoxon test, using PHARM/PCS software version 4 (MCS Consulting, Wynnewood, PA, United States). The sample and reference compound concentration ranges reported below refer to the final concentrations into the reaction mixture, which did not show any interference at the characteristic wavelengths of the tests carried out.

#### Antioxidant and Free-Radical Scavenging Activities

##### 2,2-Diphenyl-1-Picrylhydrazyl Assay

The scavenging ability of prickly pear PEs and FEs was evaluated according to Smeriglio et al. (2017). In brief, 37.5 µl of the test sample (6.25–50 μg/ml and 15.63–125 μg/ml for PE and FE, respectively; 6.25–50 μg/ml and 62.50–500 μg/ml for betanin and indicaxanthin, respectively) was added in 1.5 ml of fresh 10^−4^ M DPPH methanol solution, shaken vigorously for 10 s, and incubated at room temperature (RT) for 20 min. The absorbance was recorded at 517 nm. Trolox (1.25–10 μg/ml) was used as the reference standard.

##### Trolox Equivalent Antioxidant Capacity Assay

The TEAC test was performed according to the method described by Smeriglio et al. (2018). In brief, a reaction mixture (1:5, *v*/*v*) consisting of 1.7 mM ABTS radical and 4.3 mM (NH_4_)_2_S_2_O_8_ was incubated in the dark at RT for 12 h, diluted with deionized water until an absorbance of 0.7 ± 0.02 at 734 nm, and used within 4 h. Fifty microliters (50 µl) of the test sample (3.125–25 μg/ml and 15.63–125 μg/ml for PEs and FEs, respectively; 1.56–12.50 μg/ml and 31.25–250 μg/ml for betanin and indicaxanthin, respectively) were added to 1 ml of the aforementioned reagent, shaken, and incubated in the dark for 6 min at RT. The absorbance was recorded at 734 nm against a blank consisting of methanol. Trolox (0.63–5 μg/ml) was used as the reference standard.

##### Ferric Reducing Antioxidant Power Assay

The FRAP test was carried out according to Barreca et al. (2016). In brief, 50 µl of the test sample (1.56–12.50 μg/ml and 6.25–50 μg/ml for PEs and FEs, respectively; 3.13–25 μg/ml and 62.50–500 μg/ml for betanin and indicaxanthin, respectively) was incubated for 4 min with 1 ml of the fresh prewarmed (37°C) reagent consisting of 300 mM buffer acetate (pH 3.6), 10 mM 2,4,6-Tris (2-pyridyl)-s-triazine (TPTZ)-40 mM HCl, and 20 mM FeCl_3_. The absorbance was recorded at 593 nm. Trolox (1.25–10 μg/ml) was used as the reference standard.

##### Oxygen Radical Absorbance Capacity Assay

The ORAC test was carried out according to Smeriglio et al. (2019). Twenty microliters of PEs (0.63–5 μg/ml), FEs (3.13–25 μg/ml), betanin, and indicaxanthin (31.25–250 μg/ml), diluted in 75 mM phosphate buffer (pH 7.4), were mixed with 117 nM fresh fluorescein solution (120 µl) and incubated 15 min at 37°C. After that, 60 µl of the fresh 40 mM AAPH solution was added by starting the reaction. The fluorescence decay was recorded every 30 s for 90 min (λ_ex_ 485 nm; λ_em_ 520 nm) using a fluorescence plate reader (Fluostar Omega, BMG labtech, Ortenberg, Germany). Trolox (0.25–2.50 μg/ml) was used as the reference standard.

#### Anti-Inflammatory Activity

##### Bovine Serum Albumin Denaturation Assay

The BSA denaturation assay was carried out according to Denaro et al. (2021). In brief, 80 µl of the test sample (15.63–125 μg/ml and 31.25–250 μg/ml for PEs and FEs, respectively; 62.50–500 μg/ml and 125–1,000 μg/ml for betanin and indicaxanthin, respectively) was added to 100 µl of 0.4% BSA fatty acid-free and 20 µl of phosphate-buffered saline (PBS, pH 5.3). The absorbance was recorded at 595 nm at the starting time (T0) and after 30 min of incubation at 70°C. Diclofenac sodium (7.81–62.50 μg/ml) was used as a positive control.

##### Protease Inhibition Assay

The anti-tryptic activity was evaluated according to Smeriglio et al. (2020). In brief, 200 μl of the test sample (7.81–62.50 μg/ml and 15.625–125 μg/ml for PEs and FEs, respectively; 31.25–250 μg/ml and 125–1,000 μg/ml for betanin and indicaxanthin, respectively) was added to the reaction mixture consisting of 12 μl trypsin (10 μg/ml) and 188 μl Tris-HCl buffer (25 mM, pH 7.5). Two hundred microliters of 0.8% casein was added starting the incubation time (20 min, 37°C). Perchloric acid (400 μl) was used as the stopping reagent. The resulted cloudy suspension was centrifuged at 3,500 × g for 10 min, and the absorbance was recorded at 280 nm. Diclofenac sodium (7.81–62.50 μg/ml) was used as the reference standard.

### Antioxidant and Anti-Inflammatory Activities on *in vitro* Cell-Based Model

#### Cell Model

Experiments were carried out on Caco-2 transwell models (CacoReady™, Readycell, Barcelona, Spain). Cells (8.5 × 10^4^ cells/cm^2^, passage number 48–58) were seeded on polyester microporous filters in 24-well HTS plates and cultured for 21 days in completed Dulbecco’s modified Eagle medium (DMEM) according to Denaro et al. (2020).

#### Cell Treatments

Before carrying out the experiments, the monolayer integrity was checked by measuring the transepithelial electrical resistance (TEER) according to Denaro et al. (2020). After a preliminary screening in a wide non-cytotoxic concentration range (0.1–100 µM), by which the optimum of antioxidant and anti-inflammatory responses was reached highlighting a dose-dependent behavior (data not shown), the antioxidant and anti-inflammatory activities of pure betalains (betanin and indicaxanthin) and betalain-rich prickly pear extracts (red and orange FEs and PEs) were evaluated on a Caco-2 transwell model in equimolar concentration (10 µM) according to Denaro et al. (2021). The aim was not only to evaluate the antioxidant and anti-inflammatory effects of pure substances and prickly pear extracts but also to experimentally verify a possible synergistic effect.

In brief, pure betalains were dissolved in DMSO and diluted in DMEM obtaining 10 µM solutions (DMSO 0.1%, *v*/*v*), whereas prickly pear extracts (FEs and PEs) were dissolved in DMSO and diluted in DMEM (DMSO 0.1%, *v*/*v*) in order to obtain 10 µM solutions of the most representative compounds (betanin in red FE and PE and indicaxanthin in orange FE and PE).

The antioxidant and anti-inflammatory activities of pure betalains and prickly pear extracts were evaluated by trigging inflammation with interleukin-1β (IL-1β) 25 ng/ml. After that, cell monolayers were treated on the apical side with 10 µM betanin, 10 µM indicaxanthin, red FE (RF, 10 µM betanin), red PE (RP, 10 µM betanin), orange FE (OF, 10 µM indicaxanthin), orange PE (OP, 10 µM indicaxanthin), and 10 µM dexamethasone (Dex) as reference anti-inflammatory drugs, whereas completed DMEM (0.75 ml) was added on the basolateral side.

DMEM containing 0.1% DMSO and 25 ng/ml IL-1β were used as negative and positive controls, respectively. The same treatments without 25 ng/ml IL-1β were carried out to evaluate the possible effects induced on the cellular monolayers by pure betalains and prickly pear extracts in the absence of the inflammatory stimulus.

Cells were incubated for 24 h at 37°C, 5% CO_2_. Supernatants were collected and stored at −80°C until the subsequent analyses. Post-quality control assays such as the TEER measurement and Lucifer yellow (LY) test were assessed in order to evaluate the Caco-2 monolayer integrity according to Denaro et al. (2020).

#### Determination of Intracellular Reactive Oxygen Species

The intracellular ROS levels were assessed according to Tesoriere et al. (2014) by recording the fluorescence resulting from the intracellular oxidation of 2′,7′-dichlorofluorescin diacetate (DCF-DA), a cell-permeable non-fluorescent probe. DCF-DA in PBS (10 µM) was added to the medium 30 min before ending the treatment of the cells to label intracellular ROS. The medium was then removed, and the cells were washed five times with 1x PBS and lysed using 500 μl of cold 0.1% Triton X-100. The resulting suspensions were diluted using 1x PBS, and the fluorescence (λ_ex_ 485; λ_em_ 535) was recorded by a plate reader (FLUOstar Omega, BMG LABTECH, Ortenberg, Germany).

#### Determination of Inflammatory Markers

Interleukin-6 (IL-6) and IL-8 release were measured by high-sensitivity human ELISA kits (Cymax IL-6 YIF-LF-EK0260 and IL-8YIF-LF-EK0262, Ab Frontiers, Adipogen Corporation, San Diego, CA, United States) according to the manufacturer’s instructions, whereas NO release was evaluated by Griess reagent, according to Tesoriere et al. (2014), by using sodium nitrite as the reference standard (1.0–15 µM).

#### Cell Viability

Cell viability was assessed by 3-(4,5-dimethylthiazol-2-yl)-2,5-diphenyltetrazolium bromide (MTT) assay according to Kenzaoui et al., 2012 before the cell treatments, to find the most appropriate concentration range to test the antioxidant and anti-inflammatory activities of prickly pear extracts and pure isolated compounds, and after IL-1β–induced inflammation.

### Statistical Analysis

Ten independent experiments in triplicate (*n* = 3) were carried out for both *in vitro* cell-free and cell-based assays. Results were expressed as mean ± standard deviation (S.D.). Data were analyzed by one-way analysis of variance (ANOVA), followed by Tukey’s test and the Student–Newman–Keuls method by SigmaPlot12 (Systat Software, Inc., San Jose, CA, United States). Results were statistically significant for *p* ≤ 0.05.

## Results and Discussions

### Extraction Yield, Phytochemical Characterization, and Isolation of Indicaxanthin

Although the presence of betalains drives to a mutual exclusion of other pigments such as anthocyanins, it is well-known that prickly pears contain other classes of polyphenols (Timoneda et al., 2019). The QuEChERS extraction method, modified according to Smeriglio et al. (2019), allowed obtaining very pure betalain-enriched extracts with a high extraction yield (27.32 ± 1.55% - 29.72 ± 1.89% for FEs and 27.90 ± 1.05% - 32.85 ± 2.25% for PEs, respectively).

**FIGURE 1:**
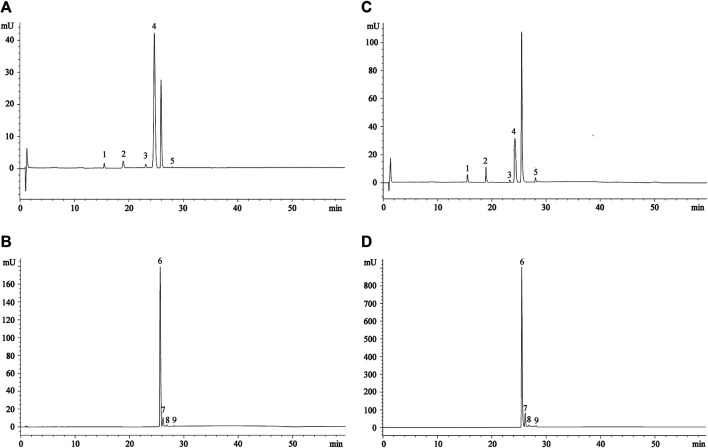
Representative chromatograms of prickly pear flesh **(A,B)** and peel extracts **(C,D**) acquired at 477 nm (betaxanthins) and 538 nm (betacyanins), respectively. Peak numbers referred to compounds shown in Table 1.

The LC-DAD-ESI-MS/MS analysis allows identifying five betaxanthins and four betacyanins in all the extracts investigated, with the exception of orange and yellow FE, in which gompherenin I and betanidin were not detected (Table 1). These results are in accordance with previous studies (Del Socorro Santos Díaz et al., 2017; Smeriglio et al., 2019), in particular with our previous study corroborating the hypothesis that for the same extraction method used, what most influences the expression of the investigated metabolites are the growth pedoclimatic conditions. Figure 1 shows the representative chromatographic profile of a prickly pear FE (Figures 1A,B) and PE (Figures 1C,D) acquired at 477 nm (betaxanthins) and 538 nm (betacyanins), respectively.

**TABLE 1:** Betalainic profile in red (R), orange (O), and yellow (Y) prickly pear flesh and peel extracts (FEs and PEs, respectively) by LC-DAD-ESI-MS/MS analysis. Results are expressed as mean ± standard deviations of ten independent experiments (*n* = 3).

Peak, n.	Compound	R_t_ (min)	λ_max_(nm)	MS (*m/z*)[M + H]^+^	MS/MS (*m/z*)[M + H]^+^		FE			PE	
								g IE^a^/100 g DE^§^			
***Betaxanthins***						**R**	**O**	**Y**	**R**	**O**	**Y**
1	Muscaaurin VII	15.78	477	349	305	0.77 ± 0.10	1.16 ± 0.17	1.01 ± 0.04	1.47 ± 0.40	2.25 ± 0.79	1.12 ± 0.17
2	Vulgaxanthin I	19.25	475	340	323	1.52 ± 0.18	2.63 ± 0.09	1.56 ± 0.29	2.49 ± 0.35	3.96 ± 0.17	1.46 ± 0.19
3	γ-aminobutyric acid-Bx	23.62	467	297	253	0.23 ± 0.06	0.82 ± 0.19	0.81 ± 0.07	1.01 ± 0.27	1.10 ± 0.09	0.79 ± 0.02
4	Indicaxanthin	24.47	486	309	265	37.60 ± 4.18	98.01 ± 9.55	78.27 ± 5.77	22.27 ± 4.50	70.46 ± 9.96	50.18 ± 6.12
5	Methionine-Bx	27.95	477	343	299	0.37 ± 0.07	0.49 ± 0.07	0.63 ± 0.05	1.42 ± 0.25	0.47 ± 0.09	0.66 ± 0.10
***Betacyanins***						**g BE°/100 g DE^§^**					
6	Betanin	25.88	538	551	389	18.94 ± 1.36	1.20 ± 0.04	1.16 ± 0.17	27.30 ± 5.85	2.45 ± 0.13	1.54 ± 0.08
7	Isobetanin	26.11	538	551	389	0.80 ± 0.12	0.08 ± 0.01	0.05 ± 0.01	1.67 ± 0.23	0.18 ± 0.04	0.09 ± 0.02
8	Gompherenin I	26.82	540	551	389	0.24 ± 0.04	n.d.	n.d.	0.74 ± 0.16	0.03 ± 0.00	0.04 ± 0.01
9	Betanidin	28.22	540	389	345	0.05 ± 0.01	n.d.	n.d.	0.21 ± 0.06	0.02 ± 0.00	0.01 ± 0.00

a IE, indicaxanthin equivalents; ^§^DE, dry extract; ^°^BE, betanin equivalents.

As can be seen already from the chromatograms (Figure 1), indicaxanthin (peak n. 4) and betanin (peak n. 6) represent the most abundant compounds. The same trend was observed in all the three colors of prickly pears investigated (Table 1). Considering this, because the indicaxanthin unlike betanin is not commercially available, a molecular exclusion chromatography starting from the indicaxanthin-rich sample (orange FE) in order to isolate this bioactive compound was carried out. Indicaxanthin was obtained with a high purity degree (≥98%) as confirmed by LC-DAD-ESI-MS/MS analysis. This allowed quantification of the betaxanthins and betacyanins content of the prickly pear extracts by expressing the results as indicaxanthin and betanin equivalents (Table 1).

The flesh extracts showed the best betaxanthin content. The orange FE showed the highest indicaxanthin content (98.01 ± 9.55 g/100 g DE), followed by yellow FE and red FE (78.27 ± 5.77 g and 37.60 ± 4.18 g/100 g DE, respectively). The same behavior was also observed for the other betaxanthins identified, with the exception of methionine-Bx, which was more expressed in yellow FE (0.63 ± 0.05 g/100 g DE), followed by orange (0.49 ± 0.07 g/100 g DE) and red (0.37 ± 0.07 g/100 g DE) ones. On the contrary, despite the orange PE being the most representative source of indicaxanthin (70.46 ± 9.96 g/100 g DE), followed by yellow PE and red PE (50.18 ± 6.12 g and 22.27 ± 4.50 g/100 g DE, respectively), according to previous results, the other betaxanthins follow a different trend in PE, with the orange one, which represents always the most important source, but followed by red and yellow ones (Table 1). The peel extracts, on the contrary, showed the best betacyanins content with the red one, which showed the best betanin content (27.30 ± 5.85 g/100 g DE), followed by orange and yellow ones (2.45 ± 0.13 g and 1.54 ± 0.08 g/100 g DE, respectively). The same trend was also observed for the other betacyanins both for PE and FE (Table 1).

Considering this, we can conclude that the betaxanthins content is from 1.3- to 2.6-fold higher in orange fruits with respect to the yellow and red ones. On the contrary, the betacyanins content is from 12.8- to 16.8-fold higher in red fruits with respect to the orange and yellow ones. Finally, the prickly pear peel, which is considered only a waste product today, is a valuable source of betalains that could be reused in the nutraceutical field in a circular economy perspective.

### Antioxidant and Anti-Inflammatory Activities by *in vitro* Cell-Free Assays

The antioxidant and anti-inflammatory activities of prickly pear extracts were first screened by different *in vitro* cell-free assays by comparing the activity of plant complexes with the most abundant pure isolated compounds (indicaxanthin and betanin) and the reference standards (trolox and diclofenac sodium for antioxidant and anti-inflammatory activities, respectively). The results of the preliminary screening are shown in Table 2.

**TABLE 2:** Antioxidant and anti-inflammatory activities of prickly pear flesh and peel extracts (FEs and PEs, respectively) in comparison with their most abundant compounds (betanin and indicaxanthin) and the reference standard (trolox and diclofenac sodium for antioxidant and anti-inflammatory activities, respectively).

IC_50_ (µg/ml) (CLs 95%)^a^						
Sample	ORAC	DPPH	FRAP	TEAC	BSA	Protease
Red FE	2.52^¥&*$§^ (2.20–2.93)	44.12^¥Φ&*$§^ (35.85–54.50)	26.91^¥&*$§^ (23.34–31.08)	43.72^¥Φ&*$§^ (35.51–53.50)	33.50^Ψ¥Φ&*$§^ (28.41–39.52)	20.53^Ψ¥Φ&*$§^ (16.72–23.84)
Red PE	2.24^¥&*$§^ (1.92–2.54)	14.03^Ψ¥Φ&*$§^ (11.92–16.64)	4.32^ΨΦ&*$^ (3.61–5.12)	9.52^ΨΦ&*$§^ (8.53–11.04)	26.12^Ψ¥Φ&*$§^ (22.33–30.74)	13.44^Ψ¥Φ&*$§^ (11.58–15.73)
Orange FE	2.50^¥*$§^ (1.47–3.33)	41.53^¥Φ&*$§^ (34.82–49.51)	22.85^¥&*$§^ (20.25–25.84)	35.42 ^¥Φ&*$§^ (29.73–42.35)	104.92^¥Φ&*$§^ (86.85–126.73)	56.48^¥Φ&*$§^ (50.66–62.27)
Orange PE	1.52^Φ&*$^ (1.34–1.94)	6.54^Φ&*$^ (5.62–7.63)	4.06^Φ&*$^ (3.33–4.92)	8.11^Φ&*$§^ (6.72–9.50)	82.55^Φ*$§^ (67.92–100.35)	37.95^Φ*$^ (29.55–44.58)
Yellow FE	3.75^&*$§^ (3.02–4.64)	69.32^&*$§^ (58.03–83.10)	38.32^&*$§^ (25.55–47.13)	79.25^&*$§^ (64.03–98.05)	163.84^&*$§^ (119.63–224.44)	77.54^&*$§^ (68.44–85.91)
Yellow PE	3.22^*$§^ (2.81–3.83)	23.42^*§^ (19.04–28.72)	6.26^*$§^ (5.52–7.18)	14.04^*§^ (12.22–16.02)	83.08^*$§^ (66.87–103.19)	42.55^*$§^ (34.46–51.27)
Betanin	10.52^*§^ (8.44–11.22)	25.42^*§^ (19.63–37.07)	11.82*§ (9.43–14.52)	15.35^*§^ (14.41–16.43)	217.46^*§^ (164.72–287.11)	158.80^*§^ (136.82–174.71)
Indicaxanthin	135.44^$§^ (111.22–164.08)	407.42^$§^ (386.08–425.64)	272.54^$§^ (257.91–289.06)	98.84^$§^ (82.54–112.24)	658.76^$§^ (627.44–686.55)	528.95^$§^ (514.28–562.42)
Reference standard	0.73 (0.35–1.73)	3.92 (1.58–6.72)	3.82 (1.75–5.48)	3.05 (2.52–3.54)	40.88 (33.59–49.33)	35.27 (29.44–40.87)

a Data were expressed as half-maximal inhibitory concentration (IC_50_, μg/ml) with confident limits (CLs) at 95% of ten independent experiments (*n* = 3); *p* < 0.001 vs. red PE; ^Ψ^ *p* < 0.001 vs. orange FE; ^¥^ *p* < 0.001 vs. orange PE; ^Φ^ *p* < 0.001 vs. yellow FE; ^&^ *p* < 0.001 vs. yellow PE; ^$^ *p* < 0.001 vs. betanin; ^*^ *p* < 0.001 vs. indicaxanthin; and ^§^ *p* < 0.001 vs. reference standard.

Results showed that PE exerted the best antioxidant activity with the following order of potency: orange > red > yellow. These results are in accordance with a previous study, which identified the prickly pear peel extracts as the strongest antioxidants (Gómez-Maqueo et al., 2019). Beyond the ORAC test, where both FEs and PEs showed similar activity with IC_50_ values almost overlapping, the antioxidant activity of PEs was 3- to 6-fold higher than that of the respective colors of FEs. However, the same behavior and order of potency were observed. Indeed, the prickly pear PEs and FEs showed the best antioxidant activity in the ORAC assay (hydrogen transfer–based assay) followed by FRAP (electron transfer–based assay), TEAC, and DPPH (both belonging to the electron and hydrogen transfer–based assays).

These results are in accordance with our previous study on Sicilian prickly pear extracts (Smeriglio et al., 2019). The orange extracts being the richest in betaxanthins, it seems plausible to speculate that these molecules are responsible for the greater antioxidant activity found. However, this is in contrast with the phytochemical characterization, which highlighted highest betaxanthins content in orange FE with respect to the orange PE. Moreover, as it can be seen from Table 2, the antioxidant activity of red PE and red FE is very similar to that of orange PE and FE. Considering this, being the red PE and FE, the richest sources of betacyanins, it is possible to hypothesize that the betacyanins have a key role in the antioxidant activity of the prickly pear extracts. Moreover, the particular behavior found for the orange extracts is the result of a synergistic effect between the betalain classes, as corroborated by the results of the pure bioactive molecules, which showed an antioxidant activity 2- to 7-fold (betanin) and 12- to 90-fold (indicaxanthin) lower with respect to the orange PE. The same behavior was also observed for the other colors of prickly pear extracts with the following order of potency: orange > red > yellow. The yellow PE is the only one, which did not show any statistical difference with respect to the betanin results in DPPH and TEAC assays, showing almost superimposable IC_50_ values (23.42 and 14.04 μg/ml vs. 25.42 and 15.35 μg/ml for yellow PE and betanin, respectively). On the contrary, all prickly pear extracts showed a statistically significant (*p* < 0.001) higher antioxidant activity than indicaxanthin and a statistically significant (*p* < 0.001) lower antioxidant activity than the reference standard (trolox), with the exception of the red and orange PEs in the FRAP assay, and orange PE in the DPPH assay (Table 2).

The different antioxidant activities found between betaxanthins and betacyanins may be ascribed to their different physical and chemical features, as already described in our previous study (Smeriglio et al., 2019). In particular, the strongest antioxidant power of betacyanins in cell-free–based assays, characterized by hydrophilic and buffered environments, is attributable to the greater hydrophilicity of these molecules than betaxanthins, as well as to the greater ability to establish hydrogen bonds.

Results of the present study are in accordance with those of Esatbeyoglu et al. (2014), which show that the antioxidant activity, mainly due to electron donator properties, may be mediated by direct free radical scavenging properties of betacyanins. Indeed, the catechol structure and the imino and tetrahydropyridine groups stabilize the system for resonance, leading to a stable carbocation upon an electron abstraction (Belhadj Slimen et al., 2017).

Moreover, through a mechanism known as hyperconjugation, betalains are able to reduce peroxyl and alkoxyl radicals, hindering the electron recovery. These antioxidant properties are affected by glycosylation, while they are positively influenced by acetylation (Belhadj Slimen et al., 2017).

Finally, thanks to their charged and ionizable groups, as well as lipophilic moieties, betalains may behave as amphiphiles at physiological pH, and they can easily interact with membranes contributing to their stabilization (Belhadj Slimen et al., 2017). These preliminary results were corroborated by the evaluation of the anti-inflammatory activity investigated by two simple *in vitro* cell-free assays: the BSA denaturation assay and the antiprotease activity test (Table 2). Protein denaturation is one of the first results of the inflammatory insult (Saso et al., 2001), and protease plays a pivotal role in several inflammation-based diseases (Oyedapo and Famurewa, 1995).

In both assays, all prickly pear extracts showed a strong anti-inflammatory activity by inhibiting, in a statistically significant manner (*p* < 0.001), the heat-induced BSA denaturation and protease activity, with the following order of potency: red > orange > yellow. According to the antioxidant results, also in this case, PEs showed the best activity. Moreover, the red PE and FE showed a statistically significant (*p* < 0.001) higher activity with respect to the reference standard diclofenac sodium (Table 2). A synergistic effect of the bioactive compounds was also experimentally demonstrated in this case because the plant complex showed an activity 2- to 12-fold and 6- to 40-fold higher with respect to betanin and indicaxanthin, respectively, in both anti-inflammatory assays (Table 2).

The activity of prickly pear extracts on some enzymes, which play a pivotal role in inflammation, has been already observed by Gómez-Maqueo et al. (2019). The authors demonstrated that whole fruits belonging to different cultivars (Morada, Vigor, and Sanguinos) exhibited moderate α-amylase inhibition and higher α-glucosidase inhibition, which suggests a possible use of such extracts in hyperglycemia management. Moreover, according to our results, Sanguinos peels presented the highest anti-inflammatory activity (Gómez-Maqueo et al., 2019).

### Evaluation of Intestinal Antioxidant and Anti-Inflammatory Activities

Once orange and red PEs (OP and RP, respectively) and FEs (OF and RF, respectively) have been identified as the most promising plant complexes in terms of bioactive compound content and biological activity, a study on an *in vitro* model of intestinal inflammation was carried out. Neither the prickly pear extracts nor the pure betalains (betanin and indicaxanthin) as well as trolox and dexamethasone (reference compounds) showed pro-oxidant and pro-inflammatory activities. Indeed, any statistically significant difference between them, and between them and the negative control (CTR−) for all the four antioxidant and inflammatory markers considered was observed (data not shown).

**FIGURE 2:**
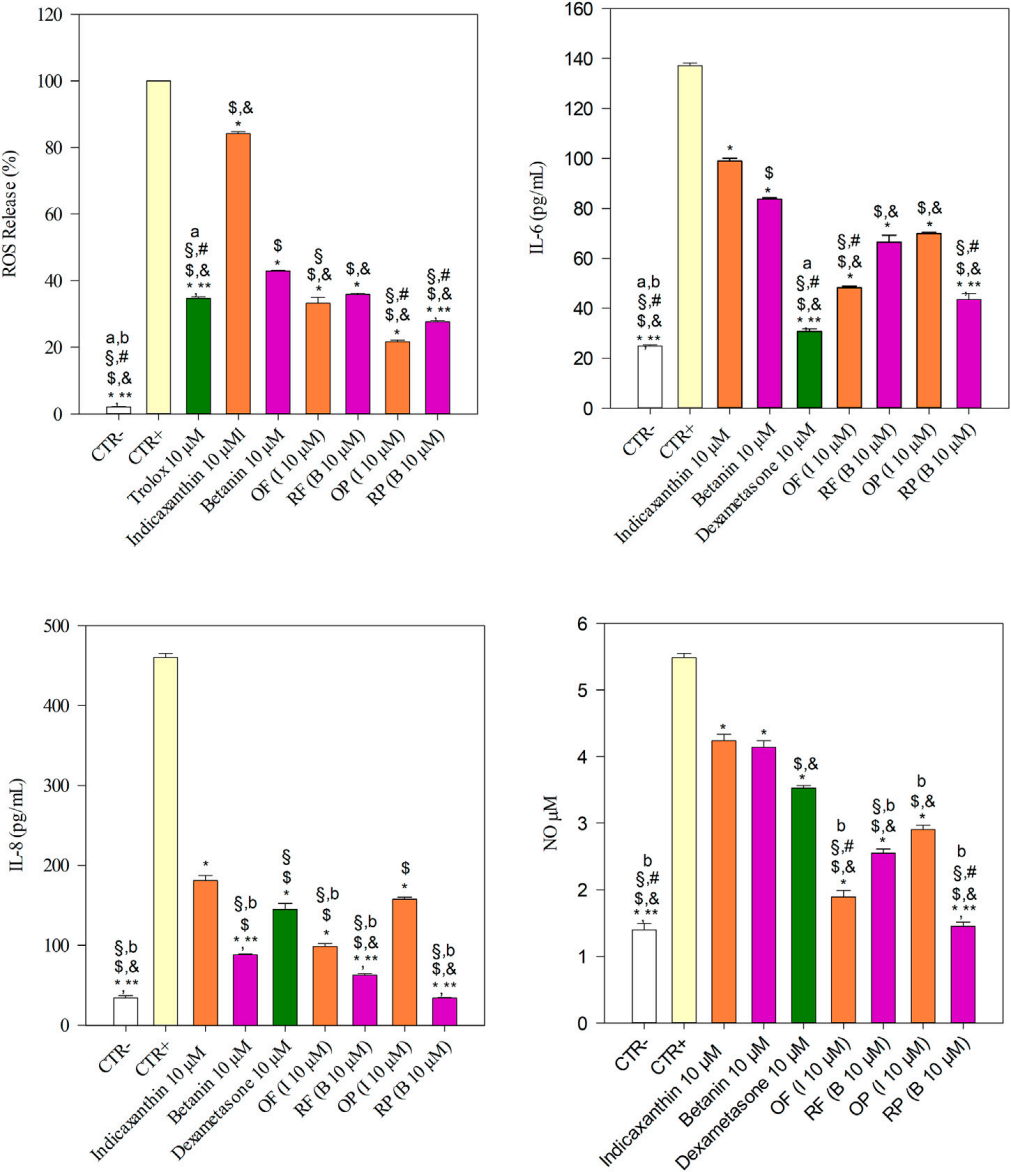
Evaluation of the antioxidant and anti-inflammatory activities of equimolar concentration (10 µM) of indicaxanthin, betanin, orange flesh and peel (OF and OP, respectively), and red flesh and peel (RF and RP, respectively) in comparison with the reference antioxidant compound (10 µM trolox) and anti-inflammatory drug (10 µM dexamethasone) by a cell-based assay carried out on a Caco-2 transwell model. IL-1β (25 ng/ml) was used to trigger the inflammation (CTR+), whereas DMEM with 0.1% DMSO was used as the negative control (CTR-). Results were expressed as ROS release percentage (%) and as concentration (pg/mL for IL-6 and IL-8, and µM for NO) of inflammatory markers released after cell treatments ±S.D. of ten independent experiments in triplicate (*n* = 3); ^*^ *p* < 0.001 vs. CTR+, ^$^ *p* < 0.001 vs. indicaxanthin, ^&^ *p* < 0.001 vs. betanin; ^§^ *p* < 0.001 vs. OP; ^#^ *p* < 0.001 vs. RF, ^**^ *p* < 0.001 vs. OF; ^a^p<0.001 vs. RP, ^b^p<0.001 vs. trolox or dexamethasone.

As depicted in Figure 2, the inflammation triggered by 25 ng/ml IL-1β resulted in a marked and statistically significant (*p* < 0.001) release of ROS and inflammatory markers investigated (∼6, ∼13, and ∼4 folds *vs.* CTR− for IL-6, IL-8, and NO, respectively), according to previous results (Denaro et al., 2020). The treatment of Caco-2 cells with 10 μM trolox, indicaxanthin, betanin, OF, RF, OP, and RP showed a statistically significant decrease (*p* < 0.001) in ROS release with respect to CTR+ (25 ng/ml IL-1β).

According to *in vitro* cell-free results, the OP showed the best free radical scavenging ability, decreasing the ROS release of 4.62-fold with respect to CTR +, followed by RP, OF, and RF (3.61-, 3.01- and 2.79-folds *vs.* CTR+, respectively). The antioxidant behavior of prickly pear extracts, with the exception of OF vs. RF, was statistically significant (*p* < 0.001).

All prickly pear betalain extracts showed a stronger and statistically significant decrease in ROS release with respect to the pure bioactive compounds (*p* < 0.001), with betanin, which showed an antioxidant power about double compared to indicaxanthin (Figure 2).

Interestingly, both prickly pear peel extracts (OP and RP) showed a strong antioxidant activity (*p* < 0.001), with respect to the reference compound (trolox) at an equimolar concentration (10 µM), whereas RF (*p* < 0.05) as well as both pure compounds (*p* < 0.001), indicaxanthin and betanin, showed a lowest and statistically significant antioxidant activity with respect to the reference compound. On the contrary, OF did not show any statistically significant difference with respect to the reference compound.

Regarding anti-inflammatory activity, the treatment of Caco-2 cells with 10 μM indicaxanthin, betanin, Dex, OF, RF, OP, and RP showed a statistically significant decrease (*p* < 0.001) in IL-6 release with respect to CTR+. Moreover, betanin showed a statistically significant decrease (*p* < 0.001) in IL-6 release compared to indicaxanthin (Figure 2).

According to this, among the prickly pear extracts, RP, which showed the highest content of betanin, showed the strongest activity followed by OF, which showed the highest content of indicaxanthin. On the contrary, RF and OP did not show any statistically significant difference. All treatments showed a statistically significant difference with respect to dexamethasone, which showed the strongest activity in decreasing the IL-6 release (Figure 2).

A different behavior has been instead observed with regard to the IL-8 and NO release (Figure 2). In the first case, dexamethasone decreased the IL-8 release only ∼3-fold with respect to the CTR+ and did not show any statistically significant difference in comparison with OP. RP showed, also in this case, the strongest activity, with statistically significant differences (*p* < 0.001) with respect to the other prickly pear extracts, followed by RF, betanin, OF, and OP, further confirming the hypothesis that betacyanins are mainly responsible for the anti-inflammatory activity of the prickly pear extracts. Finally, while analyzing the NO release, a particular behavior was observed, with any statistically significant difference between indicaxanthin and betanin. In this case, dexamethasone showed a greater activity (*p* < 0.001) than the pure molecules, whereas prickly pear extracts showed the best activity with the following order of potency: RP > OF > RF > OP (Figure 2).

Considering this, we can assume that betacyanins certainly play a pivotal role in antioxidant and anti-inflammatory activites, but a synergistic effect between betaxanthins and betacyanins exists, giving the extracts much more pronounced antioxidant and anti-inflammatory activities.

No cytotoxicity (cell viability ≥98.55% ± 2.18) or alteration of the barrier systems (TEER 800–1,000 Ω/cm^2^; LY Papp ≤9.87 × 10^–7^ cm/s and Pf ≤ 0.27%) was observed after the anti-inflammatory assays (Sun et al., 2008; Kamiloglu et al., 2015).

In the present study, as previously reported by *in vitro* and *in vivo* studies (Al-Sadi and Ma, 2007; Al-Sadi et al., 2012; Tesoriere et al., 2014), IL-1β induced an increase in intestinal epithelial tight junction (TJ) permeability. Both the prickly pear extracts and pure bioactive molecules, indicaxanthin and betanin, were able to reverse this phenomenon due to the inflammatory stimulus in accordance with a previous study carried out on the same experimental model with indicaxanthin (Tesoriere et al., 2014). This is of particular interest because it is well-known that this perturbation is due to the effect of cytokines on the structure and function of TJ (Capaldo and Nusrat, 2009; Groschwitz and Hogan, 2009), and as such, it is an important marker of therapeutic efficacy because this phenomenon is involved in intestinal inflammation, which leads to an abnormal exposure of the bloodstream to luminal substances, pathogens, and xenobiotics. This particular effect of betalains, which has been reported previously only for indicaxanthin (Tesoriere et al., 2014) and in this study for the first time also for betanin- and betalain-rich plant complexes, has not been reported for other bioactive molecules such as some flavonoids, which despite having shown a high anti-inflammatory activity, have not been able to revert the loss of epithelium integrity in inflamed Caco-2 monolayers (Sergent et al., 2010).

Although this effect must be further investigated, the physic-chemical features of betalains, which confer amphiphilic properties to them, suggest that they can be easily transported in the intestinal aqueous medium and that they can easily interact with the epithelium by directly modulating the permeability of TJ. A previous study, which has shown that indicaxanthin passively diffuses through the intestinal mucosa via the paracellular route, corroborates this hypothesis (Tesoriere et al., 2013). In the present study, the inflammation triggered by IL-1β resulted in a marked increase in the IL-6, IL-8, and NO secretion, whose releases were strongly inhibited by both pure betalains and even more by prickly pear extracts. These inflammatory mediators, which are constitutively produced by enterocytes of the intestinal mucosa, are strongly stimulated by IL-1β, a key mediator of cell communication within the inflammatory intestinal area. This led *in vivo* to the recruitment and activation, at the mucosa level, of other immune cells such as neutrophils promoting a self-sustaining inflammatory cascade. In particular, IL-8 drives the migration of polymorphonuclear cells toward the site of inflammation, whereas IL-6 cytokine stimulates the chemotaxis of neutrophils, and it is associated with colon necrosis (Martin-Viñas and Quigley, 2016). Finally, NO regulates perfusion and microvascular and epithelial permeability as well as immune responses (Kolios et al., 2004).

Even if the molecular mechanism underlying the observed anti-inflammatory effect is not within the scope of the present study, it is well-known that inflammatory cytokines activate signaling pathways that lead to the phosphorylation and inactivation of IkB, followed by the nuclear translocation of the p65/p50 dimer and its binding to specific DNA response elements, with the consequent activation of the NF-κB transcription factor, which plays a pivotal role in chronic inflammatory diseases (Kolios et al., 2004). It has been recently demonstrated that indicaxanthin inhibits the increase in the nuclear p65 subunit containing the transcriptional activation domain induced by IL-1β (Tesoriere et al., 2014). Considering that NF-κB, in synergy with other transcription factors, modulates the gene expression for inflammatory and innate immune responses as well as the genes encoding inflammatory chemokines and cytokines including IL-6, IL-8, and iNOS (Surh et al., 2001; Hoffmann et al., 2002; Hershko et al., 2004), it is possible to speculate that the anti-inflammatory activity of betalains and prickly pear extracts can be exercised through inhibition of the NF-κB activation pathway.

It has also been demonstrated in an *in vivo* model of IBD that the anti-inflammatory activity of some polyphenols is mediated by the suppression of iNOS and COX-2 as well as by the downregulation of NF-κB signaling. However, although no *in vivo* studies on the intestinal anti-inflammatory activity are yet available, it has already been demonstrated that betanin is able to revert, in a dose-dependent manner, the increase of iNOS and COX-2, NF-κB (p65) DNA-binding activity, and the serum nitrate/nitrite level in paraquat-induced kidney inflammation in rats (Tan et al., 2015). Moreover, a short-term betanin intake was able to modulate biochemical parameters reverting hepatic tissue damage induced by a hyperlipidemic diet also attenuating the oxidative stress in Wistar rats (da Silva et al., 2019).

The modulation of inflammatory processes by phytochemicals at the intestinal level may be of particular interest, although the key factor that can determine their activity is the concentration at which they reach the site of action, therefore their availability in the intestinal mucosa.

Indicaxanthin and betanin have been shown to be quite stable under digestive conditions (Tesoriere et al., 2008; Gómez-Maqueo et al., 2020). The food matrix prevented only the betanin decay at the gastrointestinal level, whereas did not affect the behavior of indicaxanthin during digestion. The main loss of betacyanins was observed during the intestinal digestion, which results in a lower bioaccessibility of betanin than indicaxanthin (10.2 ± 0.9 vs. 11.9 ± 1.1 µM, respectively, for a red medium fruit) (Tesoriere et al., 2008). These data have also been confirmed by subsequent studies (Gómez-Maqueo et al., 2020; Montiel-Sánchez et al., 2021). The authors observed that despite low pH in the gastric phase, betalains remain quite stable during this digestion step with indicaxanthin, which showed the highest bioaccessibility at the intestinal level (68.2–70.4% vs. 38.6–54.1% for betanin) (Gómez-Maqueo et al., 2020; Montiel-Sánchez et al., 2021).

Considering this, the concentration that we used to evaluate the antioxidant and anti-inflammatory activities of pure bioactive molecules and prickly pear extracts (10 µM) could reflect perfectly the *in vivo* scenario at the intestinal level. Moreover, the transepithelial transport of these two betalains across Caco-2 cells was investigated (Tesoriere et al., 2013). Substantially, they were absorbed through a paracellular route, remaining metabolically unchanged. The plant matrix did not affect the transepithelial passage of indicaxanthin but decreased the absorption rate of betanin, whose absorption is already limited by the multidrug-resistant protein 2 (MRP2)–mediated efflux. Although this event decreases the bioavailability of betanin and therefore can be seen as a negative event, paradoxically, it determines a greater availability of betanin in the intestinal mucosa, where it exerts local intestinal anti-inflammatory activity, with even more pronounced effects than indicaxanthin according to our results. Given the interest in the identification of natural compounds for the prevention and/or progression of inflammatory disorders, the results of the present study concerning a dietary highly bioaccessible compounds may offer interesting perspectives for a nutraceutical approach to intestinal inflammation.

## Conclusion

Prickly pear represents a valuable source of betalains, molecules which possess very interesting biological activities such as antioxidant, cytoprotective, antiangiogenic, and anti-inflammatory activities. Although several studies investigated the *in vitro* antioxidant and anti-inflammatory properties of prickly pear extracts or pure betalains, this is the first study which investigated selective betalin extracts and which experimentally demonstrated a synergistic activity of prickly pear extracts with respect to main bioactive compounds (indicaxanthin and betanin). Plant complexes obtained from orange and red fruits exert the strongest antioxidant and anti-inflammatory activities in the *in vitro* cell-free assays, with the red and orange peels, which showed the most interesting results, as also corroborated by the Caco-2 model in which they were able to counteract the inflammation induced by IL-1β with similar or greater effects than dexamethasone, which is used as the reference anti-inflammatory drug. These results give first evidence that prickly pear betalain-rich extract, also obtained from waste products (fruit peel), could represent a new promising nutraceutical strategy for intestinal inflammation. However, this being a preliminary study based on *in vitro* cell-free and cell-based models, the translation of results to the complex *in vivo* scenario must be done very carefully. Further *in vivo* and clinical studies should be performed in order to investigate in deep the anti-inflammatory properties of these plant complexes as well as the molecular mechanisms and cellular targets involved, which could justify the potential role of prickly pear extracts for IBD management.

## Data Availability

The raw data supporting the conclusion of this article will be made available by the authors, without undue reservation.
